# Combined in-depth, 3D, en face imaging of the optic disc, optic disc pits and optic disc pit maculopathy using swept-source megahertz OCT at 1050 nm

**DOI:** 10.1007/s00417-017-3857-9

**Published:** 2017-12-14

**Authors:** Josef Maertz, Jan Philip Kolb, Thomas Klein, Kathrin J. Mohler, Matthias Eibl, Wolfgang Wieser, Robert Huber, Siegfried Priglinger, Armin Wolf

**Affiliations:** 1 Augenklinik der Ludwig-Maximilians-Universität München, Campus Innenstadt, Mathildenstraße 8, D-80336 Munich, Germany; 2 Lehrstuhl für BioMolekulare Optik, Fakultät für Physik, Ludwig-Maximilians-Universität München, Munich, Germany; 3 Institut für Biomedizinische Optik, Universität zu Lübeck, Lübeck, Germany

**Keywords:** En face imaging, Optical coherence tomography, Swept-source OCT, Megahertz OCT, 3D rendering, Optic disc, Optic disc pit, Optic disc pit maculopathy

## Abstract

**Purpose:**

To demonstrate papillary imaging of eyes with optic disc pits (ODP) or optic disc pit associated maculopathy (ODP-M) with ultrahigh-speed swept-source optical coherence tomography (SS-OCT) at 1.68 million A-scans/s. To generate 3D-renderings of the papillary area with 3D volume-reconstructions of the ODP and highly resolved en face images from a single densely-sampled megahertz-OCT (MHz-OCT) dataset for investigation of ODP-characteristics.

**Methods:**

A 1.68 MHz-prototype SS-MHz-OCT system at 1050 nm based on a Fourier-domain mode-locked laser was employed to acquire high-definition, 3D datasets with a dense sampling of 1600 × 1600 A-scans over a 45° field of view. Six eyes with ODPs, and two further eyes with glaucomatous alteration or without ocular pathology are presented. 3D-rendering of the deep papillary structures, virtual 3D-reconstructions of the ODPs and depth resolved isotropic en face images were generated using semiautomatic segmentation.

**Results:**

3D-rendering and en face imaging of the optic disc, ODPs and ODP associated pathologies showed a broad spectrum regarding ODP characteristics. Between individuals the shape of the ODP and the appending pathologies varied considerably. MHz-OCT en face imaging generates distinct top-view images of ODPs and ODP-M. MHz-OCT generates high resolution images of retinal pathologies associated with ODP-M and allows visualizing ODPs with depths of up to 2.7 mm.

**Conclusions:**

Different patterns of ODPs can be visualized in patients for the first time using 3D-reconstructions and co-registered high-definition en face images extracted from a single densely sampled 1050 nm megahertz-OCT (MHz-OCT) dataset. As the immediate vicinity to the SAS and the site of intrapapillary proliferation is located at the bottom of the ODP it is crucial to image the complete structure and the whole depth of ODPs. Especially in very deep pits, where non-swept-source OCT fails to reach the bottom, conventional swept-source devices and the MHz-OCT alike are feasible and beneficial methods to examine deep details of optic disc pathologies, while the MHz-OCT bears the advantage of an essentially swifter imaging process.

**Electronic supplementary material:**

The online version of this article (10.1007/s00417-017-3857-9) contains supplementary material, which is available to authorized users.

## Introduction

Pits of the optic nerve head (ODP) are round or oval cavities or depressions in the optic disc and were first described by Wiethe in 1882 [1]. ODPs can either show a rather generally excavated structure or appear as a localized pit-like invagination in the optic disc. ODPs can be congenital or acquired, occur equally in women and men, and their prevalence is supposed to increase with age [2, 3]. Congenital ODPs are mostly situated in the temporal or inferotemporal segment of the optic disc. Acquired ODPs often develop centrally, adjacent to the main vessel trunk. ODPs are a rare ophthalmologic finding. Information about ODP-incidence differs widely between approximately 1 in 500 [3] to 1 in 11,000 [4, 5]. ODPs can remain clinically asymptomatic in many cases, though visual field defects can be very serious, representing full blindness in the extreme case. In 25% to 75% patients with ODPs develop optic disc pit related maculopathy (ODP-M) with retinoschisis, atrophy of inner retinal layers, serous macular detachment and significant loss of vision [2, 6, 7]. In general reattaching the retina by surgical means remains challenging, though some success has been reported for surgical solutions [8]. From histopathological point of view, an ODP is a herniation of dysplastic retinal tissue into a collagen rich excavation that often extends into the subarachnoid space through a defect in the lamina cribrosa [9]. Before the development of OCT it has not been possible to image comprehensively into ODPs in vivo. The recent progress in OCT technology has enabled researchers to gain detailed images of tissues located beneath the retinal pigment epithelium (RPE). A new and promising technology is megahertz ultra-widefield swept-source optical coherence tomography (SS-MHz-OCT). Our system uses a 1050 nm swept-source Fourier domain mode locking (FDML) OCT running at 1.68 MHz A-scan rate covering approximately 60 degrees field of view, recently reported by Mohler et al. [10–13]. While some attention has been paid to gather information about static features of ODPs recently, en face MHz imaging and virtual 3D-reconstructions of ODPs are a new approach to clarify the loco-regional structures inside the optic disc, the pit, and the appendant ODP-M [8]. Thus, the purpose of this study was to examine the optic disc and ODPs by combined in-depth , en face imaging using SS-MHz-OCT at 1050 nm, and to compare the findings to different imaging technologies.

## Methods

### Study population and 1050 nm MHz-OCT system

In this pilot study, we assessed the potential of high-resolution combined in-depth , en face imaging of the optic disc and ODPs using a SS-MHz-OCT at 1050 nm that has been developed for research purposes. En face MHz datasets and virtual 3D-reconstructions of optic discs were computed and evaluated in six patients (three women and three men, mean age 43 years, ranging from 18 to 65 years) with different types of ODPs and two further subjects including an eye with glaucoma and a healthy eye. An ophthalmic 1050 nm SS-OCT system was used to record densely sampled, isotropic wide-field volumetric datasets. The key component of this system is a rapidly wavelength-tunable FDML laser [14–16]. It operates at an ultrahigh A-scan rate of 1.68 MHz. A more detailed description of our scientific FDML MHz-OCT system can be found in references [10–12, 14, 17, 18].

The system achieves an axial resolution of ~14 μm in tissue with a sweep bandwidth of ~65 nm centered at 1050 nm. The transverse optical resolution of the system is 21 μm [18] (1/e spot size on the retina). The power incident on the cornea was 1.6 mW resulting in a shot noise limited sensitivity of 90 dB. This complies with the European Norm (EN 60825) and also with the American National Standard Institute (ANSI) standards for safe ocular exposure [19].

Apart from MHz-OCT imaging all patients also underwent clinical ophthalmologic examination including measurements of refraction, best-corrected visual acuity, slit lamp biomicroscopy, as well as funduscopic evaluation, fundus autoflourescence, infrared-imaging, OCT and EDI-OCT (Heidelberg Engineering GmbH, Germany), OPTOS imaging (Optos plc Queensferry House, Carnegie Campus, UK) and a Zeiss Fundus camera (FF 450, Carl Zeiss Jena GmbH, Germany) for photo-documentation. The diagnosis of ODPs was based on clinical history and characteristic fundus findings. None of the six eyes with ODPs had disc cupping due to glaucoma. None of the patients showed optic disc coloboma. Pathological myopia was not present in any of the patients. Though Pat. 6 showed significant synchisis, all subjects had clear enough optic media and good fixation during MHz-OCT examination, which allowed generating high-definition en face images and 3D rendering of the intrapapillary structures. Only one eye per subject was included to avoid any intra-individual correlations.

### Acquisition of depth-resolved, en face images of the optic disc and virtual 3D-reconstructions of optic disc pits

**Fig. 1:**
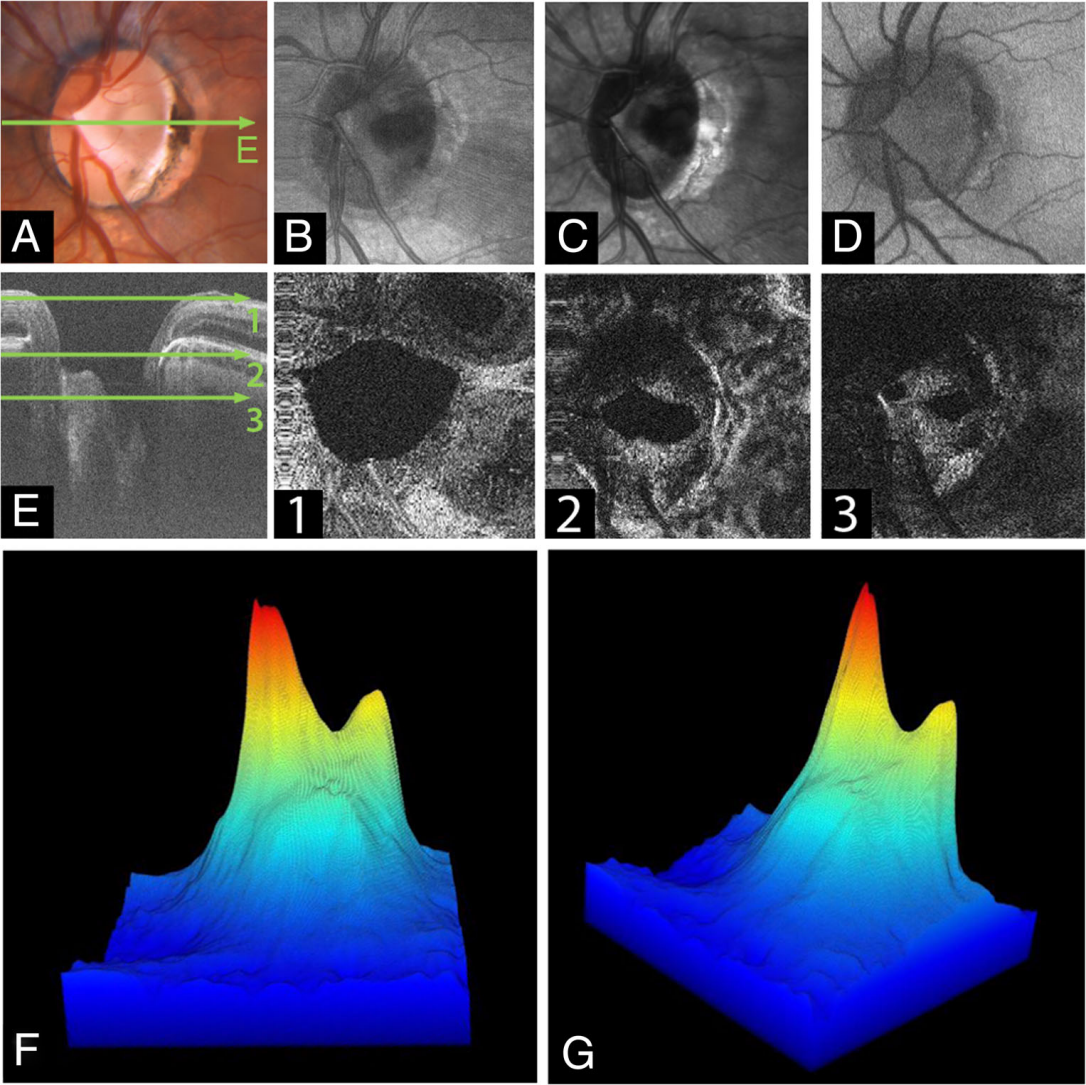
Imaging modalities of the optic disc pit (ODP). **a** Fundus photograph of the left eye of an 18-year-old man (Pat. 1 in Table 1) showing a grayish temporal ODP with adjacent hyperpigmentation and visible zone-β with parapapillary atrophy of the RPE and choriocapillaris. Green arrow indicates OCT-level of 1E. **b** En face MHz-OCT image of the optic disc. The hatchy area infero-temporal suggests detachment of neuroretinal layers. **c** Infrared image of the optic disc and ODP. **d** OPTOS autofluorescence-image of the optic disc**. e** MHz-OCT scan of the ODP. The scanned en face levels in (1–3) are shown as labeled long green arrows in **(e)**. **e1** Orifice level of the optic disc’s NFL. Superficial retinal vessels are visible. **e2** Choroidal level of the ODP. **e3** Lamina cribrosa level showing the two peaked ODP as two rather ellipsoid dark holes that break through the lamina cribrosa. **f** / **g** Rotated virtual 3D volume of the ODP, illustrating the 3-dimensional structure of the optic disc

The imaging procedure is described in reference [10] and different imaging modalities and data analysis is illustrated in Fig. 1 for a patient with ODP and ODP-M. The patients were imaged with a scanning protocol taking 2.18 s, covering 45° while consisting of 1600 × 1600 A-scans. The resulting sampling density of 8.4 μm/A-scan compared to the transversal optical resolution of 21 μm corresponds to an oversampling by a factor of 2.8. In post processing, a moving average of 4 B-frames was computed from the multi-gigabyte datasets. The region of the optical nerve head (ONH) was selected and within this volume the inner limiting anatomical structures were detected. This was performed with customized software, whereas the segmentation was based on various parameters adjusted by a trained observer for the individual B-frames. Based on the temporal position of the RPE, the 3D-reconstructions were color coded in depth; volume and maximum depth was computed. The creation of a virtual 3D reconstruction including volume and depth measurement takes 1–4 h depending on the shape of the ONH and other pathologies. The accuracy of the algorithm is estimated to be ~50 μm for finding the inner limiting interface in a single A-scan.

## Results

### Patient characteristics

Numeration of the patients refers to Table 1. The demographic and clinical characteristics of the six patients with ODPs are shown there. Upon presentation patients aged between 18 and 65 years (mean age of the sampled patients: 43.5 years ± standard deviation (SD) of this group: 19.6 years). Three patients were male, three patients were female. Bilateral ODPs were found in all male patients. All female patients presented with clinically apparent unilateral ODPs, but in two of the three the contralateral partner eye showed a deep pit-like depression (Pat. 3, Pat. 6). None of the patients presented with evident choroidal, or optic disc coloboma, and none of the eyes showed spherical equivalent (SE) of myopia more than −6.25 diopters, with an SE average of −1.2 ± 3.3 diopters (Table 1). The visual acuity at presentation ranged between 1.5 [logMar] and 0.10 [logMar] with a mean visual acuity of 0.6 ± 0.6 [logMar]. Intraocular pressure (IOP) ranged between 12 mmHg and 18 mmHg in all patients with a mean IOP of 15 ± 2.2 mmHg (Table 1). Two eyes of two patients had a history of pars plana vitrectomy (PPV) to treat macular detachment due to ODP-M (Pat. 2, Pat. 3). These eyes underwent PPV, peeling and subthreshold endolaser coagulation near the temporal margin of the optic disc with gas tamponade due to serous detachment of the macula at the age of 26 (Pat. 2 twice) or 54 (Pat. 3). Surgery failed to achieve significant visual improvement or reattachment of the retina in these cases. At the time of MHz analysis, both patients still showed persistent retinoschisis and macular detachment in the affected eyes due to ODP-M.

**Table 1:** Clinical characteristics of patients with ODPs

Patient Characteristics and Clinical Findings												
Patient No.	Age [a]	Sex	Eye	Refractive Error [dpt]	Visual Acuity [logMar]	Location of Optic Disc Pit	Number of Optic Disc Pits	Choroidal Coloboma	Previous Vitrectomy for Macular Detachment	Present Macular Detachment	Present Retinoschisis	IOP [mmHg]
1	18	m	L	0.25	0.20	temporal	1	no	no	yes	yes	14
2	26	f	R	−1.25	1.20	temporal	1	no	2× [26a]	yes	yes	16
3	55	f	R	−6.25	1.50	temporal	1	no	1× [54a]	yes	yes	14
4	65	m	R	−3.50	0.50	central	1	no	no	yes	yes	18
5	35	m	R	0.25	0.20	central	1	no	no	no	no	12
6	62	f	L	3.25	0.10	temporal	1	no	no	no	no	17
MHz-OCT Findings												
Patient No.	Retinal Tissue Herniation into Pit	Proliferation within Pit / Disc	Septum Traversing Pit	Cavity within optic nerve	Lamina Cribrosa Discontinuity	Lamina Cribrosa Displacement to Contralateral Side of Pit	Maximum Depth [μm] Measured by MHz-OCT from RPE-level	Volume [mm^3^] Measured by MHz-OCT	Diameter of Pit Opening Measured by EDI-OCT	Vertical Diameter of Optic Disc [μm] Measured by EDI-OCT	Horizontal Diameter of Optic Disc [μm] Measured by EDI-OCT	Vitreoretinal Traction in Optic Disc
1	yes	yes	yes	yes	yes	yes	618	0.2451	702	2270	1821	yes
2	yes	yes	yes	yes	yes	yes	1805	0.9185	815	2231	2529	yes
3	yes	yes	yes	yes	yes	yes	> 487		558	2165	2028	yes
4	yes	yes	no	yes	yes	uncertain	> 451		708	1338	1451	yes
5	yes	yes	yes	yes	yes	no	657	0.7824	1103	1792	1593	no
6	yes	yes	yes	yes	uncertain	uncertain	491	0.2870	865	1733	1644	yes

*[a]* age in years, *[dpt]* diopters, *m* male, *f* female, *R* right, *L* left, *OCT* optical coherence tomography, *IOP* intraocular pressure

### Optic disc pit characteristics

Most ODPs were located temporally (four cases). Two pits were located in the center of the optic disc. Every optic disc presented only one ODP. The average pit diameter at the pit-opening was 792 ± 185 μm as measured by EDI-OCT as distance from intrapapillary nerve fiber layer (NFL) to opposite NFL on level of the pit opening. The maximum depth of the ODPs measured by MHz-OCT ranged between 451 up to 1805 μm, depending on the shape of the pit. EDI-OCT could not identify the bottom of the pit in patient 2, whereas MHz-OCT was still able to detect the bottom of this very deep ODP, ranging almost 2 mm deep (Fig. 4). MHz-OCT analysis and 3D-volumetric presentation showed that ODPs manifest themselves with a high variability concerning their shape and structure (Figs. 1 and 4). Four patients presented with macular detachment (ODP-M) and retinoschisis (Table 1; Pat.1, 2, 3, 4). All patients showed herniation of retinal tissue into the pit. Also, intrapapillary proliferation was found in all patients (Table 1). Septal structures traversing the ODP were a common finding (Table 1; Pat 1, 2, 3, 5, 6). All six patients showed formation of cavities in the course of the optic nerve. But as these cavities had no obviously visible connection to the optic disc lumen or the ODP lumen, they were not pictured in the 3D volume renderings of the ODPs (Fig. 4). Upon MHz analysis all patients showed alterations of lamina cribrosa structures, including lamina cribrosa discontinuities and displacement of the lamina cribrosa to the contralateral side of the ODP.

### Multimodal imaging of ODP and ODP-M

**Fig. 2:**
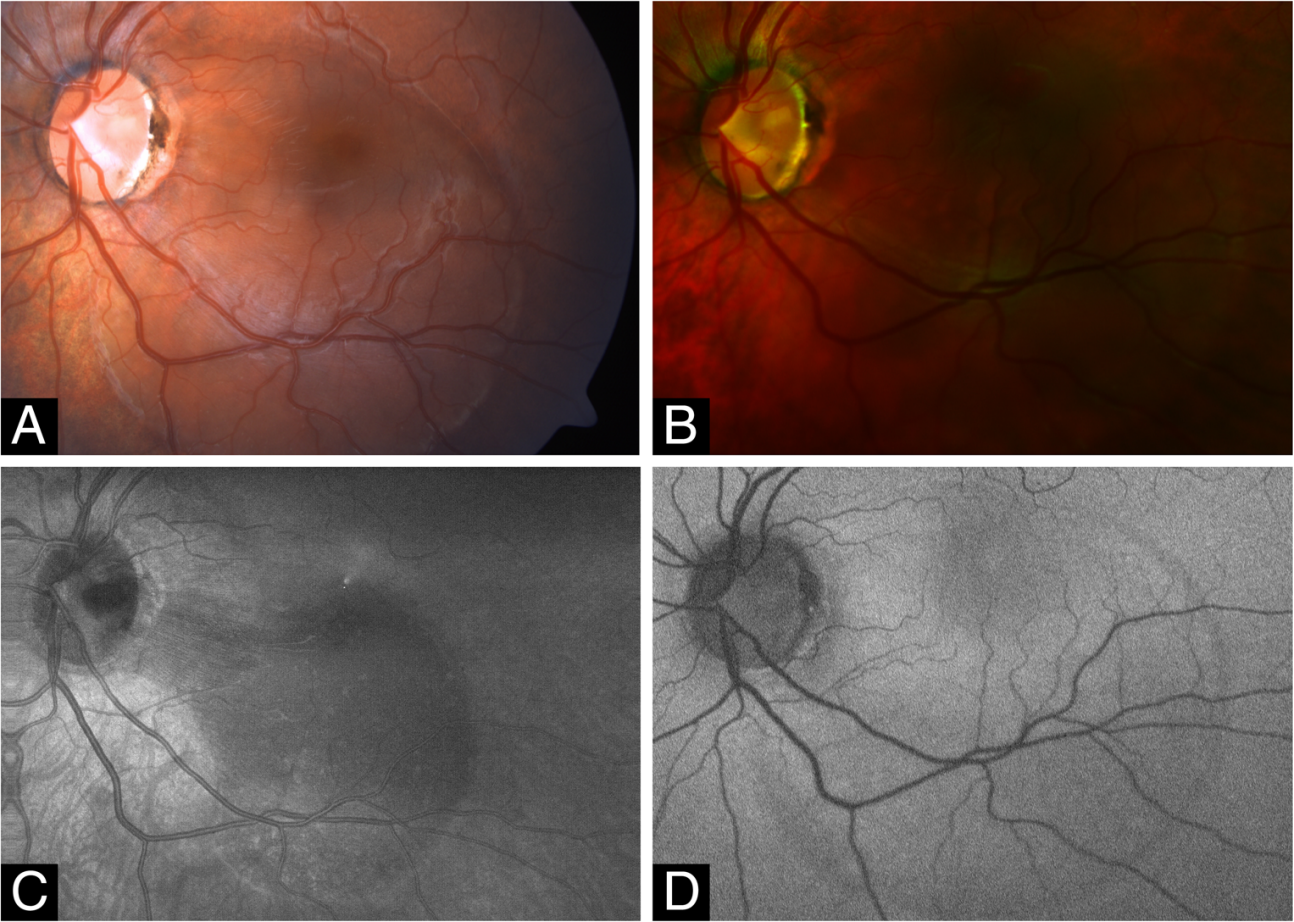
Optic disc pit maculopathy (ODP-M). **a** Fundus photograph of the left eye of an 18-year-old man (Pat. 1 in Table 1) showing a grayish temporal ODP with adjacent hyperpigmentation and detachment of the posterior pole retina, including the foveal region. **b** OPTOS pseudo-color image of the posterior showing retinal detachment. **c** En face MHz-OCT image of the posterior pole, showing neuroretinal detachment, suggesting the ODP as source of subretinal fluid. **d** OPTOS autoflourescence image of the posterior pole. While the ODP is hardly visible, the margin of the neuroretinal detachment contrasts as a darker circular lesion

**Fig. 3:**
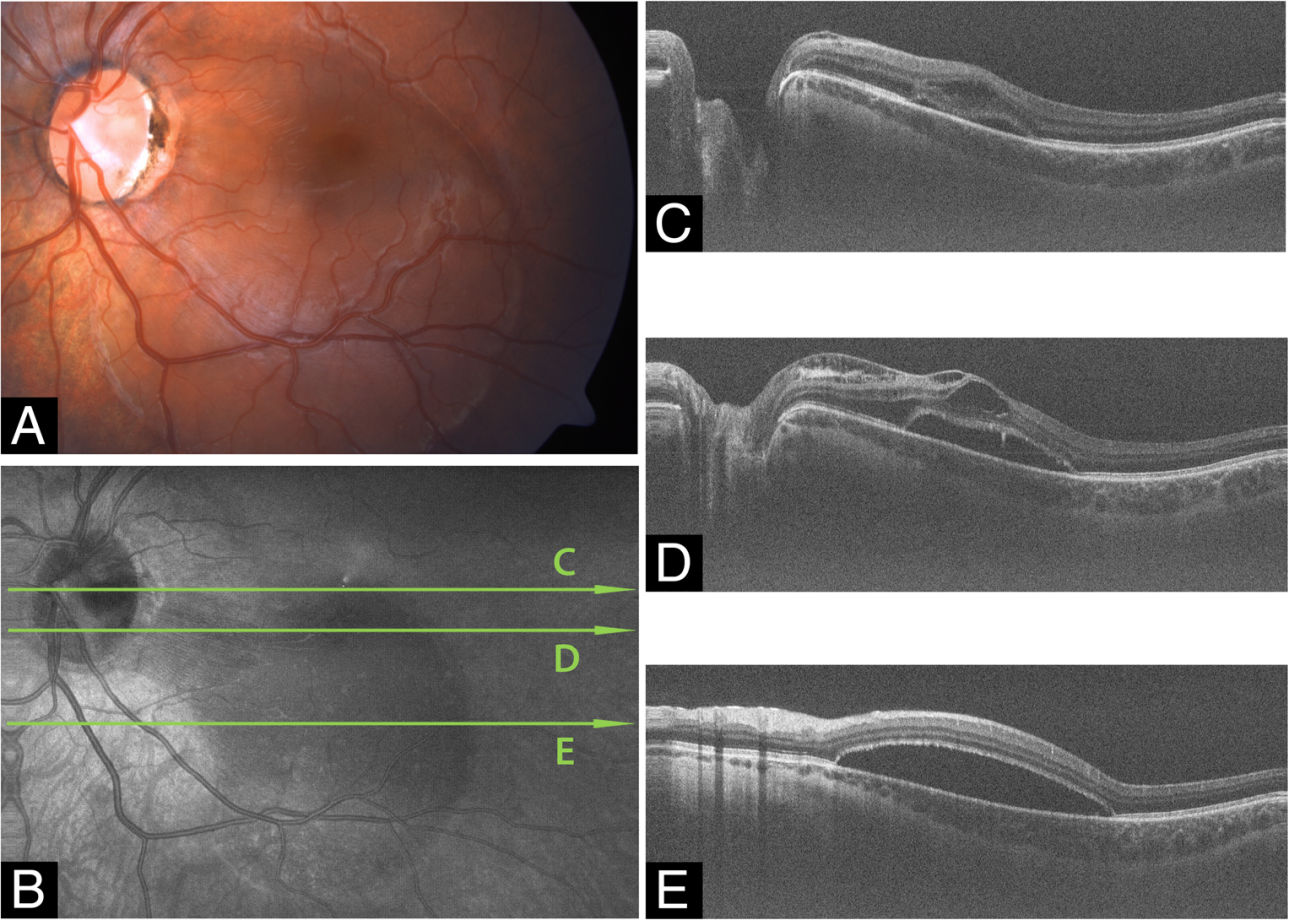
Optic disc pit maculopathy (ODP-M) on MHz-OCT. Fundus photograph, comprehensive en face MHz-OCT reconstruction and three serial sections through the ODP and adjacent retina by MHz-OCT showing the course of the pit cavity along the outer border of the pre- and retrolaminar optic nerve and all pathologic characteristics of ODP-M (retinoschisis, atrophy of inner retinal layers, serous macular detachment, intrapapillary proliferation) **(a)** Fundus photograph of the left eye of an 18-year-old man (Pat. 1 in Table 1) **(b)** Comprehensive en face MHz-OCT image showing ODP-M. The scanned OCT-lines in (C-E) are shown as labeled long green arrows in **(b)**. **c** MHz-OCT scan with temporal ODP and several schisis cavities between different retinal layers and accumulation of subretinal fluid. **d** MHz-OCT scan cutting the macula-area, with ILM as roof of the schisis cavity. More schisis cavities in the adjacent outer retinal layers including schisis within the subinternal limiting membrane space, ganglion cell layer, inner nuclear layer, outer nuclear layer, and the subretinal space. **e** Subpapillary MHz-OCT scan with retinal detachment and subretinal fluid inferior to the fovea. (BCVA 0.6 [dec] (0.2 logMar))

Covering up to 45 degrees field of view MHz-OCT generated a scan that allowed simultaneous evaluation of all ODP-associated pathologies within the optic disc as well as the macula region (Figs. 2 and 3). Different imaging modalities show different pathologic aspects in patients with ODPs (Fig. 1). On fundus photography classic congenital ODPs present mostly as grayish lesions within the surface of the optic disc, commonly located near the temporal margin of the optic disc [7, 20]. Especially in younger patients (Pat. 1, 18a; Pat. 2, 26a) with congenital ODPs, the MHz en face summation images showed very dark and circumscribable ODPs. En face MHz-OCT imaging is in this respect equal to Heidelberg infrared imaging of the optic disc that also presents the ODP as dark lesion in the optic disc surface. Especially in these two imaging modalities, horizontal ODP dimensions seemed more defined than in autofluorescence images. En face MHz-OCT proved itself as a means to depict the peripapillary choroid, with the deeper choroidal vessels forming a net around the optic disc (Fig. 1). ODP-M with retinoschisis, atrophy of inner retinal layers and serous macular detachment [2, 6, 7] can be visualized in top-view images by fundus photography, OPTOS imaging, autofluorescence and en face reconstructions. The area of retinal detachment can be seen on fundus photography, as well as on OPTOS pseudo-color images of the posterior pole. But the margin of the area of retinal detachment is better presented by autofluorescence and en face imaging. In these imaging modalities the margin of the detached retinal tissue is contrasting well with the non-detached areas (Fig. 2). In the MHz-OCT en face images the structure of the adjacent retina temporal to the ODP furthermore suggested retinoschisis with the ODP as source of the subretinal fluid. Conventional long horizontal MHz-OCT scans selected from the isotropic 1600 × 1600 A-scan dataset in post processing can show the course of the pit cavity along the outer border of the pre- and retrolaminar optic nerve and all characteristics of ODP-M, such as intra- and subretinal fluid, retinoschisis within ganglion cell layer, inner or outer nuclear layer and intrapapillary proliferations (Fig. 3).

### 3D-optic disc pit volume reconstructions

**Fig. 4:**
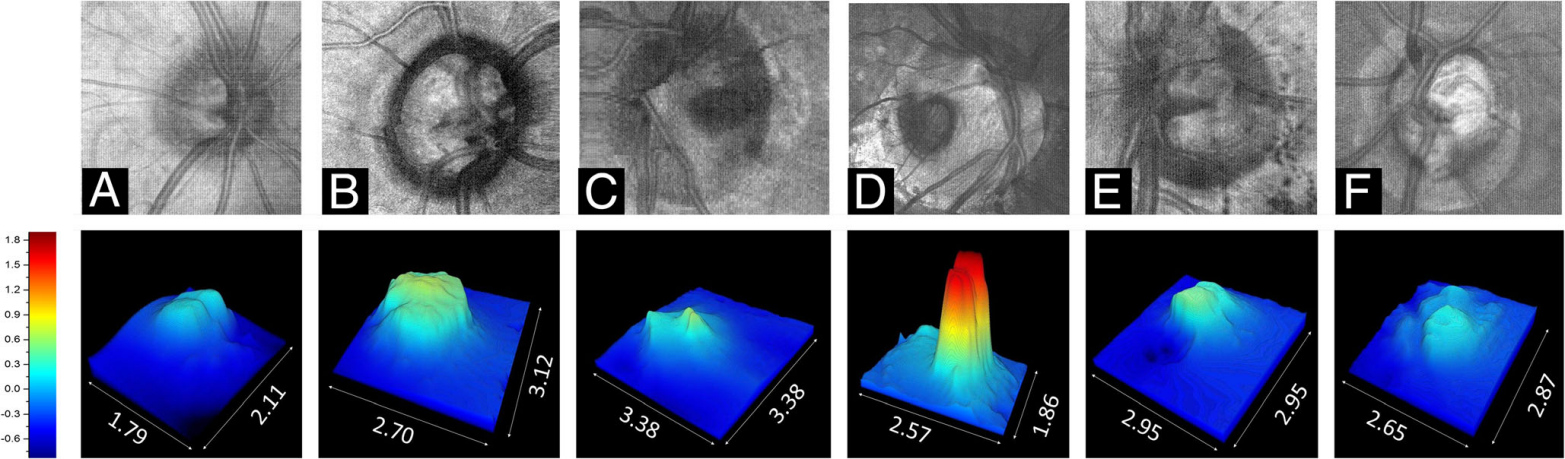
Optic disc pit MHz-OCT. Top row shows reconstructed en face MHz-OCT images of the optic disc of patients with variously shaped ODPs, one glaucomatous optic disc and a healthy control. Bottom row shows the margins of the segmented areas [mm]. The maximum depth of the optic disc/ODP measured by MHz-OCT is indicated by a color scale. Reference level [0.0] is the respective RPE level in each patient. Volume of the optic disc-cast is shown in brackets. **a** En face MHz-OCT image of the right eye of a healthy control showing a regular optic disc and flat volume cast [Volume: 0.0418 mm^3^/depth: 198 μm]. **b** En face MHz-OCT image of the right eye of a 35-year-old man (Pat. 5 in Table 1) showing a deep optic disc with central ODP adjacent to the main vessel trunk [Volume: 0.7824 mm^3^ /depth: 657 μm]. **c** En face MHz-OCT image of the optic disc in a left eye of an 18-year-old man with a classic congenital ODP (Pat.1 in Table 1). Structural 3D-analysis shows an ODP exhibiting two depth peaks [Volume: 0.2451 mm^3^ /depth: 618 μm] **(d)** En face MHz-OCT image of the optic disc in a right eye of a 26-year-old woman (Pat. 2 in Table 1). Conventional EDI-OCT failed to measure the depth of the ODP, whereas it was still possible measuring the ODP using MHz-OCT. Structural 3D-analysis shows two depth peaks [Volume: 0.9185 mm^3^ /depth: 1805 μm]. **e** En face MHz-OCT image of the optic disc in a left eye of a 62-year-old woman with dense synchisis and ODP (Pat. 6 in Table 1). Structural 3D–analysis shows an ODP exhibiting its maximum depth-peak alongside the temporal margin of the optic disc [Volume: 0.2451 mm^3^ /depth: 618 μm]. **f** En face MHz-OCT image of the optic disc in a left eye of an 81-year-old man with glaucomatous optic disc cupping. Structural 3D–analysis shows its maximum depth-peak close to the main vessel trunk [Volume: 0.103 mm^3^ /depth: 277 μm]

Conventional and en face MHz-OCT images showed ODPs with varying structure. For three-dimensional rendering of the lumen of ODPs the maximum depth of the optic disc and the ODPs measured by MHz-OCT is indicated by a color scale. Reference level [0.0] is the respective RPE level in each patient. As optic discs in patients with ODPs tend to be larger and deeper than regular optic discs, also the volume inside the optic disc is generally larger in patients with ODPs. 3D-rendering and a unique illustration-approach of the ODP-Volume as 3D-cast, facilitates gathering aspects of size, shape and structure in ODPs all at once (Fig. 4 and Table 1, suppl. videos). Classic congenital ODPs manifest themselves as rather double peaked deep temporal pits appearing dark, and very defined and circumscript on en face MHz-OCT (Fig. 4c, d, e). Acquired ODPs rather manifest close to the main vessel trunk [3] (Fig. 4b). In the optic disc in a left eye of a 26-year-old woman EDI-OCT (Spectralis Heidelberg) failed to measure the depth of the ODP, whereas it was still possible to measure the ODP using in-depth MHz-OCT. Also, this classic and very deep congenital ODP shows two depth peaks [Volume: 0.9185 mm^3^ / depth: 1805 μm] (Fig. 4d). The glaucomatous sample showed typical optic disc cupping. [Pat. characteristics: 81a, male, left eye, spherical equivalent (SE): −0.75 dpt, visual acuity (VA): 0.4 [dec] / 0.4 [logMar], IOD 28 mmHg] In this eye the respective 3D-volume reconstruction shows its single depth peak close to the main vessel trunk [Volume: 0.103 mm^3^ / depth: 277 μm]. Acquired ODPs occur in this location in aged patients with glaucoma, while the classically described temporal ODP is the most uncommon morphologic subtype found in older populations [3].

## Discussion

In this pilot study, we presented in-depth 3D, en face imaging of the optic disc and ODPs with an ultrafast A-scan rate of 1.68 MHz. To the best of our knowledge, this is the first demonstration of isotropic combined in-depth 3D, en face volumetric imaging of the optic disc and ODPs using 1050 nm MHz wide-field imaging. Though there are studies also employing SS-OCT [8, 20], the scientific MHz-device used in this study bears several advantages. The ultrafast scan rate [20]) allows for a very short sampling procedure, facilitating quick image acquisition especially in agitated patients without losing valuable information. Whereas standard spectral domain OCT systems have typical imaging ranges of ~1.5 mm in water, the reported MHz-OCT features a 2.7 mm imaging range in water. This allows visualizing even very deep ODPs (Fig. 4d), that could not be imaged with a standard spectral domain OCT. Whereas slower OCT systems only can image the small ONH area in high resolution at a time, the MHz-OCT system used in this study is able to keep exceptionally dense transversal (X,Y) sampling while imaging an area with 45° of view. Covering 45° field of view with a high sampling density, MHz-OCT generated a scan that allowed simultaneous evaluation of all ODP-associated pathologies within the disc, as well as the macula and vitreopapillary region (Figs. 2 and 3) [10–12].

In their publication Ohno-Matsui et al. describe a scanning protocol using 256 × 256 A-Scans for 3D volumetric data which were acquired in 0.8 s [20]. In our setting 1600 × 1600 A-scans for 3D volumetric data (thus covering a wider area) were acquired in 2.18 s. If the system of our study would have been used for the same scanning protocol as in Ref. 20, the scanning time would have been lower than 0.06 s. This considerable reduction regarding the scanning time of 0.06 of the MHz-OCT compared to 0.8 s with the SS-OCT [20] bears an advantage, e.g. agitated patients.

As the primary goal of this study was to assess the ODP and the appending maculopathy at the same time, a 45° field of view comprising 1600 × 1600 A-scans was chosen. We argue that it is clinically important to image the whole extent of the ODP, and the closest vicinity to the SAS, as well as the site of intrapapillary proliferation, which are situated at the bottom of the ODP. This is more important the bigger and deeper the ODP actually is. The close vicinity to the SAS can have an impact on CSF influx and diffusion into the ODP, and intrapapillary proliferation could influence the course of the appending ODP-Maculopathy. In this respect the imaging area has to be enlarged, if the ODP and the ODP-Maculopathy shall be examined at the same time.

The exact mechanism of ODP-M is still unclear, but it seems that a large variety of factors – including a putative channel-formation to the subarachnoidal space (SAS) as well as vitreo-papillary interactions may play a role. Therefore, it is important to not only image the optic disc pit, but also the surrounding structures. The study shows that ODPs manifest themselves with a high variability concerning their shape and depth (Figs. 1 and 4). There seems not to be a specific shape bearing a higher risk of ODP-M.

As MHz-OCT enables high definition, widefield three-dimensional rendering of larger areas around the optic disc at dense sampling, any tissue pathology in the neighboring regions can also be reliably assessed (Figs. 2 and 3).

Like Brown and Shields, we were also unable to visualize a putative connection between the subarachnoid space and the subretinal fluid via the ODP in en face or conventional MHz-OCT imaging. Still, MHz-OCT could show hyporeflective spaces with spotted or trabeculary hyperreflective structures near the bottom or the wall of the pit, suggestive for the retrobulbar SAS. However, these seem to be independent from the shape of the ODP in 3D reconstruction. As degradation and liquefaction of the vitreous occurs in old age [21, 22], fluid from the SAS remains subject of debate in children and adolescents with ODP-M. MHz-OCT can assess details from tissues of the ODP-bottom and the SAS. It is known that cerebrospinal fluid dynamics between the intracranial space and the SAS of the optic disc can lead to an optic nerve compartment syndrome [23]. This mechanism and the immediate vicinity of ODP and SAS could facilitate diffusion or influx of cerebrospinal fluid (CSF) from the SAS into the ODP, creating a separation of retinal layers and ODP-M. Further supporting evidence that CSF can migrate from the subarachnoid space to the sub- and intraretinal space via the ODP and vice versa is given by human case studies that showed migration of gas [24] or silicone oil [25] into the subarachnoidal space after vitreoretinal surgery. Recently it was shown that ongoing intrapapillary proliferation changes the aspect of the ODP over time and that ODP-M seems to accelerate this process or vice versa [26]. Thus, development of intrapapillary proliferations could influence the prognosis of ODP-M. As this tissue primarily occurs at the bottom of the ODP it is crucial that the whole depth of the optic pit can be imaged. Especially in cases with very deep congenital pits, where conventional OCT fails to reach the bottom, MHz-OCT can be beneficial.

In our study we could demonstrate numerous interconnected schisis cavities in various layers of the retina (Fig. 2), and we believe that fluid can migrate via these schisis cavities. Thus our MHz-OCT findings support a variation of Lincoff’s pattern [27] for the development of ODP-M (Fig. 3). Seen as disease of the vitreoretinal interface [8, 28] in ODP-M a contraction of vitreous fibers could lift and separate the herniated dysplastic retinal tissue inside the pit. This notion has been supported with the fact that MHZ-OCT-imaged intrapapillary proliferations can change significantly over time [26, 29] and seem to correlate with the severity of ODP-M The separation of incarcerated tissue allows an influx of fluid from the pit, leading to a schisis-like inner layer separation [30], which produces a mild centrocoecal scotoma. The liquefied vitreous could be a very likely source for this fluid [31]. Secondly an outer layer hole, often close to the macula, develops beneath the schisis cavity, causing a dense central scotoma. Subsequently an outer layer detachment evolves [2, 27]. This process has also been described for very shallow pits and for membranes spanning over a glaucomatous optic disc [30, 32].

An important advantage of an ultra-high speed OCT compared to lower speed OCT systems is the possibility to extract a virtual 3D reconstruction of the optic disc and different en face images from the same densely-sampled 3D-OCT dataset, facilitating direct point-to-point correlation of features of interest. In contrast, lower speed OCT systems often use a second imaging modality like a scanning laser ophthalmoscope to generate the en face images. However, using a second imaging device makes the correlation with the original OCT dataset more inaccurate. Even though the generation of depth resolved en face images directly from the OCT data would also be possible in these cases, it would feature a significantly lower resolution than the SLO image bearing the possibility of missing important details. The MHz-OCT system presented in this study overcomes this problem by its very dense isotropic sampling. The 3D volume reconstruction facilitates gathering aspects of size, shape and structure in ODPs all at once. In 3D video reconstructions also changes in intrapapillary proliferations can be visualized, especially when intrapapillary proliferations change and affect the structure of the lumen in ODPs and the optic disc over time [26, 29].

We could demonstrate in this study that a single densely and isotropically sampled 3D dataset covering a wide field of view of 45° (1600 × 1600 A scans) provides significantly more information than a standard 20° OCT dataset. Different ODP characteristics such as pit depths and size or intrapapillary proliferations could be interpreted more easily as the B-scans can be supplemented with high-resolution en face images and virtual 3D renderings.

Lower speed instruments are also capable to generate wide-field imaging, but with reduced A-scan sampling density. Therefore, one runs into a potential risk of missing fine details using too sparse sampling especially in deeper tissue layers and the ONH region.

As known from other widefield imaging modalities, vignetting and shadowing caused by the pupil and eye lashes can become a challenge with an increasing field of view [33]. Datasets acquired with a 45° protocol show no or very little shadowing. The region of the ONH and macula is rarely affected, but if scanning protocols with larger fields of view were of interest a more careful alignment strategy is beneficial [34]. Mhz-OCT imaging of ODP and cases of ODP-M failed to identify a clear mechanism in this study; however, confirmed several theories as mentioned. We cannot rule out that a combination of all mechanisms or all mechanisms per se allow the development of ODP-M. Further research is, therefore, warranted.

However, this study suggests that the wealth of information provided by densely-sampled in-depth MHz-OCT datasets is helpful for early detection of intrapapillary proliferations occurring at the bottom of ODPs and that the simultaneous acquisition of 3D-volume rendering of the ONH-area and depth-resolved en face images aids diagnosis. 3D-rendering of the optic disc pits shows that they manifest themselves with various patterns and shapes. Especially as MHz imaging generates high resolution images of retinal pathologies associated with ODP-M and allows visualizing of OPDs with depths up to 2.7 mm, MHz-OCT imaging is a beneficial method to examine deep-tissue details of optic disc pathologies such as ODPs or ODP-M.

## Electronic supplementary material

Reconstructed animation movies using MHz-OCT datasets of the optic nerve head region to supply a 3D - 360° view of the conformation within the optic disc and optic disc pits.

ESM 1 ONH_Movie_healthy_fin. Reconstructed 3D–animation of the region of the optic nerve head using a MHz-OCT-dataset of the right eye of a healthy control showing a regular optic disc and flat volume-cast [Volume: 0.0418 mm^3^ / depth: 198 μm]. (AVI 3237 kb)

ESM 2. ONH_Movie_Pat1_fin. Reconstructed 3D–animation using the MHz-OCT-datset of the optic disc in a left eye of an 18-year-old man with a classic congenital ODP (Pat.1 in Table 1). The 3D–volume-cast shows an ODP exhibiting two depth-peaks [Volume: 0.2451 mm^3^ / depth: 618 μm] (AVI 3148 kb)

ESM 3. ONH_Movie_Pat2_fin. Reconstructed 3D–animation using the MHz-OCT-dataset of the optic disc in a right eye of a 26-year-old woman (Pat. 2 in Table 1). EDI-OCT (Spectralis Heidelberg) failed to measure the depth of the ODP in this case, whereas it was still possible measuring the ODP using in-depth MHz-OCT. Also this classic congenital ODP shows an ODP-Volume cast exhibiting two depth-peaks [Volume: 0.9185 mm^3^ / depth: 1805 μm]. (AVI 2294 kb)

ESM 4. ONH_Movie_Pat5_fin. Reconstructed 3D–animation using the MHz-OCT-dataset of the right eye of a 35-year-old man (Pat. 5 in Table 1). The animation shows a deep optic disc with central ODP adjacent to the main vessel trunk, with an increased volume in the respective optic disc-cast [Volume: 0.7824 mm^3^ / depth: 657 μm] (AVI 4234 kb)

ESM 5. ONH_Movie_Pat6_fin. Reconstructed 3D–animation using the MHz-OCT-dataset of the optic disc in a left eye of a 62-year-old woman with dense synchisis and ODP (Pat. 6 in Table 1). The animated 3D–volume-cast shows an ODP exhibiting its maximum depth-peak alongside the temporal margin of the optic disc [Volume: 0.2451 mm^3^ / depth: 618 μm]. (AVI 4446 kb)

ESM 6. ONH_Movie_glaucomatous_fin. Reconstructed 3D–animation using the MHz-OCT-dataset of the optic disc in a left eye of an 81-year-old man with optic disc cupping due to Glaucoma. The animated 3D–volume-cast shows its maximum depth-peak close to the main vessel trunk [Volume: 0.103 mm^3^ / depth: 277 μm]. (AVI 2200 kb)

## Abbreviations

ANSI: American National Standard Institute
CSF: Cerebrospinal fluid
EDI-OCT: Enhanced depth imaging optical coherence tomography
FDML: wavelength-tunable Fourier-domain mode-locked laser
FML-OCT: Fourier domain mode locking optical coherence tomography
ILM: Internal limiting membrane
IOP: Intraocular Pressure
LMU: Ludwig-Maximilians-Universität
MHz-OCT: Megahertz-optical coherence tomography
NFL: Nerve fiber layer
ODP: Optic disc pit
ODP-M: Optic disc pit associated maculopathy
ONH: Optical nerve head
PPV: Pars Plana Vitrectomy
SAS: Subarachnoid space
SE: Spherical equivalent
SS-OCT: Swept-source optical coherence tomography
VA: Visual acuity

## References

[1] Wiethe T (1882). Ein Fall von angeborener Deformität der Sehnervenpapille. Arch Augenheilkd.

[2] Georgalas I, Ladas I, Georgopoulos G, Petrou P (2011). Optic disc pit: a review. Graefe's Arch Clin Exp Ophthalmol Albrecht Graefes Archiv Klin Exp Ophthalmol.

[3] Healey PR, Mitchell P (2008). The prevalence of optic disc pits and their relationship to glaucoma. J Glaucoma.

[4] Wang Y, Xu L, Jonas JB (2006). Prevalence of congenital optic disc pits in adult Chinese: the Beijing eye study. Eur J Ophthalmol.

[5] Kranenburg EW (1960). Crater-like holes in the optic disc and central serous retinopathy. Arch Ophthalmol.

[6] Christoforidis JB, Terrell W, Davidorf FH (2012). Histopathology of optic nerve pit-associated maculopathy. Clin Ophthalmol.

[7] Brown GC, Shields JA, Goldberg RE (1980). Congenital pits of the optic nerve head. II Clinical studies in humans. Ophthalmology.

[8] Yokoi T, Nakayama Y, Nishina S, Azuma N (2016). Abnormal traction of the vitreous detected by swept-source optical coherence tomography is related to the maculopathy associated with optic disc pits. Graefe's Arch Clin Exp Ophthalmol Albrecht Graefes Archiv Klin Exp Ophthalmol.

[9] Ferry AP (1963). Macular detachment associated with congenital pit of the optic nerve head. Pathologic Findings in Two Cases Simulating Malignant Melanoma of the Choroid. Arch ophthalmol.

[10] Reznicek L, Klein T, Wieser W, Kernt M, Wolf A, Haritoglou C, Kampik A, Huber R, Neubauer AS (2014). Megahertz ultra-wide-field swept-source retina optical coherence tomography compared to current existing imaging devices. Graefe's Arch Clin Exp Ophthalmol Albrecht Graefes Archiv Klin Exp Ophthalmol.

[11] Klein T, Wieser W, Eigenwillig CM, Biedermann BR, Huber R (2011). Megahertz OCT for ultrawide-field retinal imaging with a 1050 nm Fourier domain mode-locked laser. Opt Express.

[12] Mohler KJ, Draxinger W, Klein T, Kolb JP, Wieser W, Haritoglou C, Kampik A, Fujimoto JG, Neubauer AS, Huber R, Wolf A (2015). Combined 60 degrees wide-field choroidal thickness maps and high-definition En face vasculature visualization using swept-source megahertz OCT at 1050 nm. Invest Ophthalmol Vis Sci.

[13] Reznicek L, Kolb JP, Klein T, Mohler KJ, Wieser W, Huber R, Kernt M, Martz J, Neubauer AS (2015). Wide-field megahertz OCT imaging of patients with diabetic retinopathy. J Diab Res.

[14] Huber R, Wojtkowski M, Fujimoto JG (2006). Fourier domain mode locking (FDML): a new laser operating regime and applications for optical coherence tomography. Opt Express.

[15] Huber R, Adler DC, Srinivasan VJ, Fujimoto JG (2007). Fourier domain mode locking at 1050 nm for ultra-high-speed optical coherence tomography of the human retina at 236,000 axial scans per second. Opt Lett.

[16] Srinivasan VJ, Adler DC, Chen Y, Gorczynska I, Huber R, Duker JS, Schuman JS, Fujimoto JG (2008). Ultrahigh-speed optical coherence tomography for three-dimensional and en face imaging of the retina and optic nerve head. Invest Ophthalmol Vis Sci.

[17] Klein T, Wieser W, Reznicek L, Neubauer A, Kampik A, Huber R (2013). Multi-MHz retinal OCT. Biomed Opt Exp.

[18] Kolb JP, Klein T, Kufner CL, Wieser W, Neubauer AS, Huber R (2015). Ultra-widefield retinal MHz-OCT imaging with up to 100 degrees viewing angle. Biomed Opt Exp.

[19] ANSI (2000) Safe use of Lasers & Safe use of optical fiber communications. In: Institute ANS (ed) Z136 committee. American National Standards Institute—American National Standard for Safe Use of Lasers, Z136.1. 2000 (National Laser Institute, 2000)

[20] Ohno-Matsui K, Hirakata A, Inoue M, Akiba M, Ishibashi T (2013). Evaluation of congenital optic disc pits and optic disc colobomas by swept-source optical coherence tomography. Invest Ophthalmol Vis Sci.

[21] Kampik A (2012). Brief overview of the molecular structure of normal and aging human vitreous. Retina.

[22] Walton KA, Meyer CH, Harkrider CJ, Cox TA, Toth CA (2002). Age-related changes in vitreous mobility as measured by video B scan ultrasound. Exp Eye Res.

[23] Killer HE, Jaggi GP, Flammer J, Miller NR, Huber AR, Mironov A (2007). Cerebrospinal fluid dynamics between the intracranial and the subarachnoid space of the optic nerve. Is it always bidirectional?. Brain: J Neurol.

[24] Johnson TM, Johnson MW (2004). Pathogenic implications of subretinal gas migration through pits and atypical colobomas of the optic nerve. Arch Ophthalmol.

[25] Kuhn F, Kover F, Szabo I, Mester V (2006). Intracranial migration of silicone oil from an eye with optic pit. Graefe's Arch Clin Exp Ophthalmol Albrecht Graefes Archiv Klin Exp Ophthalmol.

[26] Maertz J, Mohler KJ, Kolb JP, Kein T, Neubauer A, Kampik A, Priglinger S, Wieser W, Huber R, Wolf A (2016) Intrapapillary proliferation in optic disk pits: clinical findings and time-related changes. Retina. 10.1097/IAE.0000000000001260 10.1097/IAE.0000000000001260 27617535

[27] Lincoff H, Lopez R, Kreissig I, Yannuzzi L, Cox M, Burton T (2012). Retinoschisis associated with optic nerve pits. 1988. Retina.

[28] Gandorfer A, Kampik A (2000) [Role of vitreoretinal interface in the pathogenesis and therapy of macular disease associated with optic pits]. Ophthalmol: Z Dtsch Ophthalmol Ges 97:276–279 10.1007/s003470050526 10827464

[29] Maertz J (2015) Aspect and development of Intrapapillary proliferations in optic disc PitsEuretina 2015, Nice

[30] Hirakata A, Hida T, Ogasawara A, Iizuka N (2005). Multilayered retinoschisis associated with optic disc pit. Jpn J Ophthalmol.

[31] Hirakata A, Okada AA, Hida T (2005). Long-term results of vitrectomy without laser treatment for macular detachment associated with an optic disc pit. Ophthalmology.

[32] Takashina S, Saito W, Noda K, Katai M, Ishida S (2013). Membrane tissue on the optic disc may cause macular schisis associated with a glaucomatous optic disc without optic disc pits. Clin Ophthalmol.

[33] Neubauer A, Kernt M, Haritoglou C, Priglinger S, Kampik A, Ulbig M (2008). Nonmydriatic screening for diabetic retinopathy by ultra-widefield scanning laser ophthalmoscopy (Optomap). Graefes Arch Clin Exp Ophthalmol.

[34] Cheng SCK, Yap MKH, Goldschmidt E, Swann PG, Ng LHY, Lam CSY (2008). Use of the Optomap with lid retraction and its sensitivity and specificity#. Clin Exp Optom.

